# Editorial: Intertwining paths: sensorimotor and cognitive dynamics in neurocognitive aging

**DOI:** 10.3389/fnagi.2026.1914038

**Published:** 2026-07-09

**Authors:** Adérito Seixas, Redha Taiar, Danúbia Sá-Caputo

**Affiliations:** 1 Escola Superior de Saúde Fernando Pessoa, Porto, Portugal; 2 Université de Reims Champagne-Ardenne, MATIM, Reims, France; 3 Universidade do Estado do Rio de Janeiro, Rio de Janeiro, Brazil

**Keywords:** cognitive-motor interaction, digital biomarkers, multidomain interventions, neurocognitive aging, sensorimotor function

## Introduction

The World Health Organization defines healthy aging as developing and maintaining the functional ability that enables wellbeing in older age. That definition is especially apt for this Research Topic because function in later life is never purely cognitive and never purely motor. It emerges from the interaction of intrinsic capacity, environment, and task demands. By 2050, the global population aged 60 years and older is projected to reach 2.1 billion, with two-thirds living in low- and middle-income countries (Chen et al., 2025; World Health Organization, 2020). Against that demographic reality, the 16 papers gathered in *Intertwining Paths: Sensorimotor and Cognitive Dynamics in Neurocognitive Aging* arrive at the right time and with an important message. Neurocognitive aging is embodied. The aging brain does not decline in isolation. It declines through walking, balancing, hearing, orienting, gripping, writing, and adapting movement to ever more complex environments. The Research Topic's value lies not only in showing that motor and cognitive changes co-occur, but in showing why they co-occur and how those links may be measured, modified, and translated into practice.

A useful framework for reading this Research Topic comes from the long-standing gait-cognition literature, the motoric cognitive risk construct, and WHO's emphasis on function rather than diagnosis alone. Montero-Odasso et al. (2012) argued that gait and cognition offer a complementary window onto brain function and falling risk, while later work showed that combined motor and cognitive decline improves prediction of incident dementia relative to either alone. The present Research Topic extends that logic. It shows that the relevant network is broader than gait and executive function alone. Cerebellar processing, vestibular integration, hearing, strength, neural oscillations, handwriting, and dual-task performance all belong within the same explanatory field. In this sense, the Research Topic moves the field away from a narrow search for single markers and toward a systems account in which declines in executive control, sensory integration, postural adaptation, and neural efficiency manifest as ecologically meaningful failures in everyday action.

The neurophysiology papers are particularly helpful in building that systems account. Song et al. show that the relationship between muscle strength and cognition in cognitively impaired older adults is partly mediated by working memory and resting-state EEG, suggesting that grip strength may act as a behavioral proxy for central neural integrity. Jahanian-Najafabadi and Davoodi, in a large systematic review, map oscillatory motor-learning findings across adulthood and highlight age-related changes in alpha, beta, theta and related dynamics, together with a tendency toward broader activation patterns in older adults. Ma et al. add a cerebellar perspective by demonstrating that older adults can perform normally on the MMSE while already showing deficits on the Cerebellar Cognitive Affective Syndrome scale and slowed perceptual sensorimotor integration. Racz et al. then extend the EEG literature by proposing flatter scale-free spectral slopes as a candidate signature of altered excitatory-inhibitory balance and compensatory processing in aging. Together these papers strengthen the view that neurocognitive aging depends on distributed rather than local failure, and that sensitive physiological markers may reveal decline earlier than global screening tests do.

The Research Topic is equally strong when it turns to sensory and orientation systems. Gerb et al. show that aging, cognitive impairment, and chronic peripheral vestibular loss selectively impair horizontal, but not vertical, spatial orientation, suggesting that vestibular-cognitive aging is anisotropic and likely dependent on distinct representational strategies. Downey et al. show that hearing ability shapes both baseline dual-task performance and response to multidomain training in older adults with mild cognitive impairment, with important sex-related differences. These observations matter well beyond the specific studies. The WHO world report on hearing (Chadha et al., 2021) emphasizes the public health relevance of adult hearing screening and intervention, and the (Livingston et al., 2024) among the life-course modifiable risks for dementia while adding untreated vision loss and high LDL cholesterol. Recent cohort evidence from the Framingham Heart Study (Kolo et al., 2025) further links hearing loss with smaller brain volume, greater white matter abnormality, executive decline, and higher incident dementia risk over 15 years. Our argument is straightforward: hearing and vestibular care are not adjuncts to cognitive aging research. They are part of its core translational agenda.

On gait, balance, falls, and frailty, the Research Topic is both clinically relevant and refreshingly nuanced. Choi et al. provide a strong translational contribution by showing prospectively that anticipatory braking and lateral balance instability, derived from wearable inertial sensors, predict future falls over 24 months, with body mass index modifying the effect of braking control. Rizzato et al. show why quiet standing is not enough: older adults may appear broadly preserved on static balance while already showing marked impairment during unexpected perturbations, precisely the sort of demand that matters in real life. Song et al., studying motoric cognitive risk in rural China, reveal how chronic pain, falls, depression, social support, living conditions, vision loss, and chronic disease all cluster around motor-cognitive vulnerability. Wen et al., by contrast, show that in hospitalized older adults the usual sensorimotor-cognition associations become weaker after adjustment, with education emerging as the dominant predictor. That negative finding is useful. It reminds us that motor-cognitive metrics are context-sensitive and that acute illness, multimorbidity, and care setting can distort or suppress relationships seen in community cohorts.

The Research Topic's digital-biomarker strand is one of its most forward-looking features. Hosseini et al. show, in a systematic review and meta-analysis, that sensor-based dual-task motion analysis consistently detects slower gait, shorter steps, and less stable timing in dementia, with dual-task conditions generally amplifying group differences. Burgio et al. make a parallel argument for digital handwriting, synthesizing evidence that writing performance is a sensitive output of distributed motor-sensory-cognitive networks and that high-resolution kinematic measurement could support diagnosis, monitoring, and rehabilitation. Horváth-Pápai et al. add an unconventional but clinically attractive possibility by revisiting primitive reflexes as low-cost behavioral markers linked to cognition and modifiable by physical activity. The opportunity here is obvious: digital or low-burden markers could make embodied cognitive screening more scalable. The warning is equally obvious: standardization, external validation, and fairness remain unresolved. The dual-task sensor literature is still dominated by Alzheimer-focused cohorts, and handwriting analysis will need careful attention to language, education, device, and cultural bias before it can support equitable deployment.

Intervention evidence in the Research Topic is promising, but not yet definitive. Andrade-Guerrero et al. provide mechanistic preclinical support by showing that combined exercise improved locomotion, balance, strength, muscle atrophy, cortical amyloid burden, and mitochondrial measures in advanced 3xTg-AD mice. In humans, Piskin et al. report that a short exergaming intervention shifted EEG complexity toward a more youthful profile and was accompanied by better balance, mobility, and game performance. Pan et al. further show that immersive motor-cognitive VR may improve global cognition and frailty severity in cognitive frailty, although current evidence derives from very few trials. Downey et al. complicate the intervention picture in a useful way by showing that hearing status and sex influence training effects, while Horváth-Pápai et al. suggest that physical activity mediates the link between primitive reflexes and cognition. The broad lesson is not simply that “exercise helps.” It is that multimodal interventions are likely to work through multiple routes at once: sensory access, executive demand, motor adaptation, motivation, and neural plasticity. Future trials should therefore measure mechanism, not just outcome.

This Research Topic also exposes what the field still lacks. Many studies are cross-sectional. Some rely on small, healthy, or otherwise selected samples. Measures vary substantially, especially in EEG processing, dual-task design, and operational definitions of cognitive frailty or sensory impairment. Yet the larger gap is strategic rather than technical. The populations most likely to benefit from integrated sensorimotor-cognitive care are often those least represented in biomarker development: rural older adults, people with multimorbidity, inpatients, those with sensory loss, and residents of lower-resource settings. WHO notes that by mid-century most older adults will live in low- and middle-income countries, and that healthy aging depends on both capacities and environments. In that context, Song et al.'s rural Chinese motor-cognitive risk syndrome study is a standout contribution, but also a reminder of how much more inclusive work is needed. Multimorbidity should not simply be adjusted away as nuisance variance. It should be modeled as the very condition under which aging bodies and aging brains interact.

Overall, this Research Topic advances a practical research agenda. Older adults should be assessed dynamically, not statically; multimodally, not in silos; and in real-world terms, not solely through brief cognitive screens. Gait, balance, hearing, vestibular orientation, grip strength, handwriting and EEG are not competing biomarkers. They are complementary readouts of shared system integrity. The next step is to test them together in longitudinal, diverse, clinically embedded cohorts, and to use them to personalize interventions that combine movement, cognition, sensory rehabilitation, and supportive environments. If there is one conclusion that captures this Research Topic, it is that the path forward is not to choose between sensorimotor aging and cognitive aging. It is to understand the loops that bind them, and then to design care around those loops.

## Methodological and translational appraisal

The Research Topic's principal methodological strength is triangulation. It spans animal work, clinic-based cohorts, community samples, wearable sensing, EEG, force-platform analysis, narrative and systematic reviews, and meta-analyses. This variety matters because the sensorimotor-cognitive interface is unlikely to be captured by any single platform or level of analysis. A second strength is that some papers are explicitly mechanistic rather than merely associative, particularly the EEG mediation study, the exergaming complexity study, the vestibular orientation work, and the preclinical exercise experiment. A third strength is that the Research Topic includes both positive and context-limiting findings, which reduces the risk of a simplistic “everything correlates with cognition” narrative.

Its weaknesses are equally clear. Cross-sectional designs dominate. Small samples remain common in mechanistic studies. There is still substantial heterogeneity in task design, outcome definition, and signal-processing choices, especially in EEG and dual-task paradigms. Several intervention studies lack rigorous causal inference because they lack control groups, balanced trial designs, follow-up, or sensitive cognitive outcomes. These are not minor issues. They determine whether the field can move from plausible mechanisms to reliable prognostic and treatment tools.

Translationally, the papers support four concrete shifts. First, dynamic balance and dual-task gait should be prioritized over static or single-task assessments when the aim is fall or dementia-risk stratification. Second, hearing and vestibular assessment should be built into neurocognitive aging pathways rather than treated as specialist add-ons. Third, digital biomarkers should be developed ecologically, with emphasis on wearables, everyday movement and handwriting, but only alongside harmonized protocols and external validation. Fourth, interventions should be multimodal by design and mechanism-aware in evaluation.

The most important future direction is inclusive longitudinal integration. The field now needs cohorts and trials that repeatedly measure gait, balance, sensory function, vestibular status, grip strength, neural dynamics, and cognitive outcomes across time, and that recruit beyond healthy, urban, well-functioning volunteers. Rural populations, hospitalized adults, people with chronic pain or polypharmacy, and underrepresented regions should become central rather than peripheral to study design. That is not only an equity issue. It is also a scientific requirement if the field wants biomarkers and interventions that work outside idealized research settings.

## Conclusion

This Research Topic highlights that neurocognitive aging is best understood as an integrated, adaptive process in which neural change, sensorimotor capacity, cognition, environment and task demands continuously interact. The contributions included here show that age-related decline is not uniform and may only become evident under complex, dynamic or ecologically meaningful conditions, such as dual-tasking, perturbation, spatial orientation, gait adaptation or digital motion analysis. They also reinforce the value of combining accessible clinical measures with emerging digital biomarkers to improve early detection, risk stratification and monitoring. Importantly, the evidence points toward interventions that target coupled systems rather than isolated impairments, including physical exercise, exergaming, virtual reality, sensory optimisation and multidomain training. Future research should prioritize longitudinal, multimodal and clinically implementable designs capable of translating mechanistic insight into personalized strategies to preserve autonomy, mobility and cognitive health in aging populations.
